# Oncologic outcomes of pre- versus post-operative radiation in Resectable soft tissue sarcoma: a systematic review and meta-analysis

**DOI:** 10.1186/s13014-020-01600-9

**Published:** 2020-06-23

**Authors:** Xinmiao Yang, Lihua Zhang, Xiaojing Yang, Weiwei Yu, Jie Fu

**Affiliations:** Department of Radiation Oncology, Shanghai Jiao Tong University Affiliated Sixth People’s Hospital, No.600 Yishan Road, Shanghai, 200233 China

**Keywords:** Oncologic outcomes, Preoperative radiation, Postoperative radiation, Soft tissue sarcoma, Meta-analysis

## Abstract

**Background:**

Postoperative radiation therapy for soft tissue sarcomas demonstrated good local recurrence-free survival rates and survival outcomes. However, the results remained controversial. This study aimed to evaluate the role of preoperative and postoperative radiation therapy for the treatment of resectable soft tissue sarcomas.

**Methods:**

The electronic database PubMed, MEDLINE, Cochrane Library, and EMBASE were performed from inception till 30 November, 2019. The effect of preoperative versus postoperative radiation therapy on resectable soft tissue sarcomas was compared and then assessed.

**Results:**

A total of 15 studies with 12,813 patients were included, and most of these had acceptable quality scores. Of these, 10 studies reported data on local recurrence. The pooled results indicated no significant differences between preoperative radiotherapy and postoperative radiotherapy groups for local recurrence, with a risk ratio (RR) and 95% confidence interval (CI) of 0.84 (95%CI = 0.58–1.21). No difference was observed in the overall survival and distant metastasis between the two groups. According to the pooled results, preoperative radiotherapy group showed a significant risk for complications (RR = 2.11, 95%CI = 1.36–3.27).

**Conclusions:**

The postoperative radiation therapy does not increase the local recurrence, overall survival, and distant metastasis, but might result in lowering complications.

## Background

Soft tissue sarcomas are a heterogeneous group of malignancies that arise from the mesenchymal stem cells and are defined based on histologic subtype, tumor grade, and anatomic location [1]. Over the past several years, many advancements in the field of molecular genetics have been made to histopathologically classify soft tissue sarcomas [2]. However, the prognosis of these tumors is diversified due to over 50 histologic types [3].

For localized diseases, the treatment generally involves radical surgical excision. Function-preserving surgery in combination with pre- or postoperative radiation therapy is regarded as the current standard treatment for majority of soft tissue sarcomas [4], and is recommended according to the current European Sarcoma Network Working Group clinical practice guidelines [5]. However, due to heterogeneous histology and tumor biology of various soft tissue sarcoma subtypes, managing patients in clinical settings with soft tissue sarcomas is inherently ambiguous [6]. Moreover, recent studies have supported that without radiation therapy or postoperative radiation therapy in the extremity of sarcomas would arise good local recurrence-free survival rates [7, 8]. The combination of improved histologic analysis with new molecular diagnostics has largely enhanced our understanding with regard to characterization of tumor subtypes, providing greater insights into tumor biology [9]. With the progression of soft tissue sarcoma subtypes and advances in the individual behavior, our treatment strategies need to be refined.

In 2018, meta-analysis [10] of 16 studies revealed that the use of postoperative external beam radiation therapy (EBRT) for local tumor control in patients with resectable soft tissue sarcoma might result in positive effects on the overall survival rate. In a previous study, only 11 studies were included to compare the outcomes of preoperative and postoperative radiation therapy. However, the study is limited by the inclusion of small number of non-randomized studies, and the positive effect on overall survival only existed in the subgroup of retroperitoneal soft tissue sarcoma. Recently, many studies have been published on this topic [11–14]. Therefore, the newly published literatures make it possible to conduct a powerful and a more persuasive systematic review and meta-analysis on this topic by providing more specific evidences on whether the pre- and postoperative radiation in soft tissue sarcoma could benefit patients.

## Methods

### Literature search

A systematic literature search of the databases PubMed, MEDLINE, Cochrane Library, and EMBASE were performed by individual and a combination of search terms such as “soft tissue”, “sarcoma”, “retroperitoneal sarcoma”, “radiation”, “preoperative”, and “postoperative”. The process was conducted by following the Preferred Reporting Items for Systematic Reviews and Meta-Analyses (PRISMA) statement [15]. To include more potential literatures, the search terms used were kept as broadly as possible to identify relevant publications. The bibliographies of all relevant studies and reviews, and Google Scholar for studies that cited relevant studies were also checked to identify any other relevant publications. All publications up to 30 November, 2019 were included in this meta-analysis.

### Eligibility criteria

Studies that compared the outcomes between preoperative and postoperative radiotherapy cohorts in patients with resectable soft tissue sarcomas were included. The inclusion criteria were as follows: (1) randomized controlled trials (RCTs), other clinical trials, and observational studies comparing pre- and postoperative radiation in patients with retroperitoneal or soft tissue sarcomas; (2) the studies that reported oncologic outcomes (local recurrence and overall survival) in both groups; (3) necessary data was extracted from the original studies; (4) studies published in English; and (5) only studies that provided more detailed information were included if multiple publications were reported in duplicate sample population.

Reviews, reports, comments, case studies and case series, abstracts or posters for conferences, studies focusing on animal experiments or experiments in vitro, and studies in languages other than English were also excluded. Moreover, the study population with advanced diseases or the studies that reported only outcome measures related to wound healing and/or radiation dose were also excluded.

### Data extraction

Two investigators (*Weiwei Yu and Xiaojing Yang*) independently screened all abstracts, and included by reviewing the full-texts of the studies. The necessary information was extracted from the included studies using a customized and standardized form by the two reviewers above independently. To assess the level of agreement of the extracted data between the two reviewers, Kappa coefficient was employed and a consensus was reached on all items by the two reviewers. For each included study, the following information was extracted: the author and year of publication, country, study design, study population characteristics (e.g., age, sex, and nation), characteristics of soft tissue sarcoma (localization, grading, and resection status), type of intervention, and data on the outcomes of interest (local recurrence, overall survival, wound healing).

### Quality scoring of studies

The two reviewers (*Jie Fu and Xinmiao Yang*) independently assessed the quality of observational studies by the Newcastle-Ottawa Scale (NOS) [16], which is a procedure used for independently assessing the methodological quality of meta-analysis for case–control and cohort studies. NOS is evaluated mainly by three categories: (1) patient selection (three items); (2) comparability of the two study arms (two items); and (3) assessment of the outcomes (two items).

Studies were given a maximum of one point for each numbered item within the selection and exposure categories and a maximum of two points for comparability. Studies were graded based on an ordinal scoring scale, with the score ranging from 2 points to 9 points. A scale of 0 to 4 stars was considered to be of poor quality, 5 to 6 stars as moderate quality, and 7–9 stars as high quality.

For RCTs, the Jadad scale, also named as the Oxford quality scoring system, which is a procedure to independently assess the methodological quality of a clinical trial was used to assess the study quality [17]. The overall score ranges from 0 to 5. To set a minimum standard for the paper’s results to be included in a meta-analysis, a Jadad score of less than 3 might be used to select the studies [18].

### Statistical analysis

Combined risk ratios (RRs) and 95% confidence intervals (CIs) were calculated for the selected dichotomous outcomes by inverse variance method with random effects. For comparing preoperative versus postoperative radiotherapy on local recurrence, overall survival, and wound healing, subgroup analyses were performed for tumor localization. More stratified analyses were subsequently performed with respect to localization, grading, and resection status of the study population and outcome. Heterogeneity between the included studies was assessed using *I*^*2*^ and Q tests. Heterogeneity was defined as low, moderate, and high to *I*^*2*^ values of 25, 50, and 75%, respectively [19]. The publication bias was assessed using the Begg’s rank correlation [20] and Egger’s weighted [21] regression methods (*P* < 0.05 was considered to be statistically significant publication bias). Forest plot generation and statistical analyses were performed using RevMan. The Begg and Egger tests were employed using Stata 15.0 (Stata Corporation, TX, USA). A *P* value of < 0.05 was considered to be significant for all analyses.

## Results

### Study selection

**Fig. 1:**
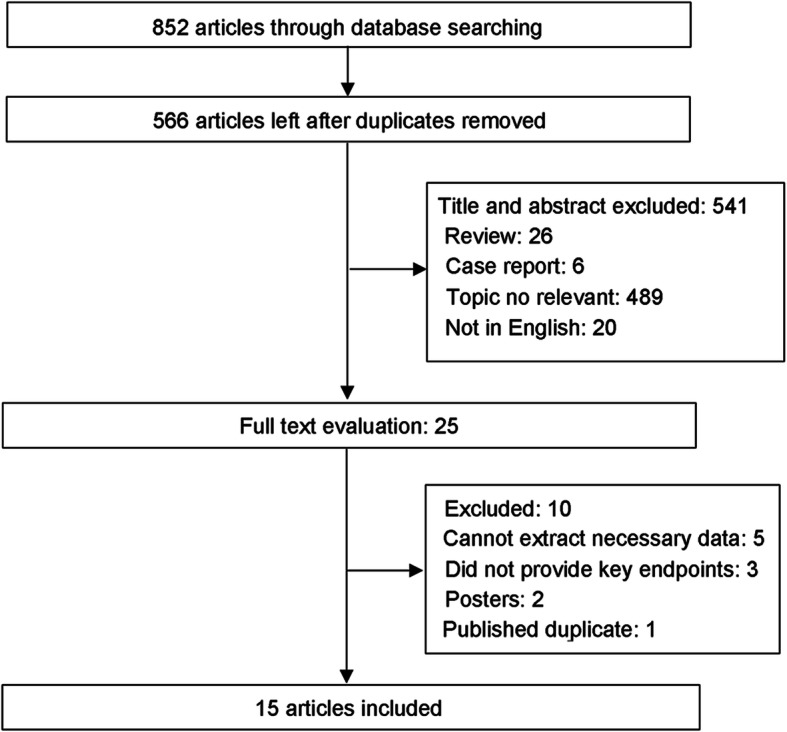
Flow chart of study selection process

The systematic literature search of databases yielded a total of 852 manuscripts. Of these, 286 were excluded due to duplications, and 541 were excluded after screening the abstracts and titles. Finally, 15 studies (1 RCT, 1 prospective study, and 13 retrospective studies) [11–14, 22–32] were included for data extraction and meta-analysis after retrieving 25 full-length manuscripts. A flow chart of study selection process was given in Fig. 1.

### Study characteristics

In total, 15 studies with 12,813 patients were included in this study and the characteristics of the included studies and participants were summarized in **Additional** Table 1 and Table 1. The included studies were published between 1982 and 2019 and the participants were recruited from 1971 to 2002. The sample size of the included studies ranged from 23 to 9604. Eight studies were conducted in United States [12–14, 22–26], two in Canada [11, 30], one in Norway [27], Egypt [29], France [31], and Italy [32]. Majority of the included studies used radiotherapy either as a preoperative or postoperative external beam radiotherapy and only three studies used intraoperative radiotherapy.

**Table 1:** Characteristics of the included studies

Study included	Study design	Sample size		Means of follow-up time (years)	Localization	Outcomes	
		PRCT	POCT			PRCT	POCT
Frezza, et al., 1982 [22]	Retrospective	50	49	4.75	Extremities, Trunk, Head/Neck	LC: 2/50 WC:4/50	LC: 5/49 WC:2/49
Suit, et al., 1985 [23]	Retrospective	60	110	1.00–11.00 ^a^	Extremities, Trunk, Head/Neck	LCO: 0.86 (0.73–0.94) ^f^ LC: 6/60 OS:37/60	LCO:0.84 (0.74–0.90) ^f^ LC: 13/110 OS:80/110
Cheng, et al.,1996 [24]	Retrospective	48	64	5.30 (1.25–16.00) ^b^	Extremities, Trunk, Head/Neck	LCO: 0.80 ± 0.12 ^g^ LC: 7/48 OS:34/48 WC:15/48	LCO: 0.91 ± 0.08 ^g^ LC:6/64 OS:35/64 WC:5/64
Pollack, et al.,1998 [25]	Retrospective	128	165	8.75	Extremities, Trunk, Head/Neck	LCO: 0.88 OS: 32/128	LCO: 0.73 OS: 10/165
O’Sullivan,et.al.,2002 [11]	RCT	88	94	3.30 (0.27–5.60) ^b^	Extremities, Trunk, Head/Neck	WC: 31/88 OS: 74/88 LR: 6/88 DM: 0/88	WC: 16/94 OS: 68/94 LR: 6/94 DM:1/94
Zagars, et al.,2003 [12]	Retrospective	271	246	6.40 (1.70–29.9)/ 9.10 (1.30–29.20) ^c^	Extremities, Trunk, Head/Neck	LR:36/271 OS: 108/271 DM: 98/271	LR: 56/246 OS: 144/246 DM: 87/234
Kuklo, et al.,2005 [13]	Retrospective	59	58	6.10/8.40 ^d^	Extremities, Trunk, Head/Neck	OS: 49/59 WC: 19/59	OS: 48/58 WC: 17/58
Schoenfeld, et al.,2006 [26]	Retrospective	7	16	11.00 (1.20–25.8) ^b^	Extremities, Trunk, Head/Neck	LR: 1/7	LR: 1/16
Jebsen, et al.,2008 [27]	Retrospective	106	356	5.00 (0.10–20.00) ^b^	Extremities, Trunk, Head/Neck	LR: 12/107 OS: 53/107	LR: 45/356 OS: 163/356
Sampath, et al.,2011 [28]	Retrospective	293	528	5.25 (0.00–17.00) ^b^	Both	LCO: 0.93 OS: 190/293	LCO: 0.87 OS: 317/528
El-Sayed, et al.,2012 [29]	Prospective	24	39	3.92 (0.50–5.50) ^b^	Extremities, Trunk, Head/Neck	LCO: 0.87 LR: 2/24 OS: 21/24 WC: 6/24	LCO: 0.76 LR: 6/39 OS: 31/39 WC: 3/39
Moore, et al.,2014 [30]	Retrospective	122	62	10.00	NA	WC: 29/122	WC: 5/62
Toulmonde, et al.,2014 [31]	Retrospective	13	62	6.50	Retroperitoneum	LR: 0/13 OS: 1/13	LR: 33/62 OS: 45/92
Lazarev, et al.,2017 [14]	Retrospective	2358	7246	6.90	Extremities, Trunk, Head/Neck	OS: 1542/2358	OS: 5166/7246
Greto, et al.,2019 [32]	Retrospective	19	72	4.10 (0.10–17.70) ^b^	Extremities, Trunk, Head/Neck	LR: 5/15 OS: 15/19 DM: 10/19	LR: 14/72 OS: 38/72 DM: 33/75

*Abbreviations*: *PRCT* preoperative radiotherapy, *POCT* postoperative radiotherapy, *OS* overall survival, disease-free, *DFS*, *LC* local recurrence, Local control *LCO*, *WC* wound complication, *DM* distant metastasis, *NA* not available

^a^, range of follow time

^b^, means and range of follow time

^c^, means and range of follow time for preoperative and postoperative, respectively

^d^, means of follow time for preoperative and postoperative, respectively

^f^, 95% confidence interval

^g^, strand deviation

### Quality assessment of studies

Most of the included studies had an acceptable quality, and the overall quality scores were presented in **Additional Table****2**. One study designed as RCT was evaluated for full scores by the Jadad scale [11], and the other studies were had 6–8 points by NOS. None of the included RCTs were assessed to have a low quality.

### Local recurrence

**Fig. 2:**
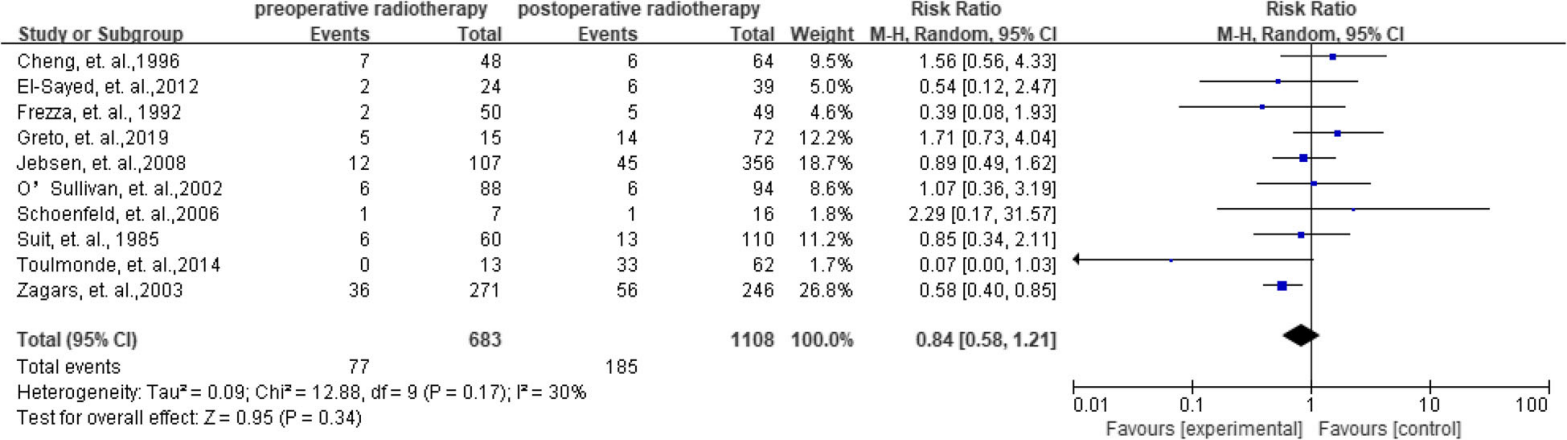
Summarized local recurrence

As shown in Figs. 2**,** 10 studies with 683 preoperative radiotherapy patients and 1108 postoperative radiotherapy patients reported the data on local recurrence. The RRs of the individual studies ranged from 0.37 to 2.50. When the RRs were summarized together, the results showed an acceptable heterogeneity (*I*^2^ = 35%). The risk of local recurrence between preoperative and postoperative radiotherapy showed no difference with a pooled RR as 0.84 (95%CI = 0.58–1.21).

### Complications

**Fig. 3:**
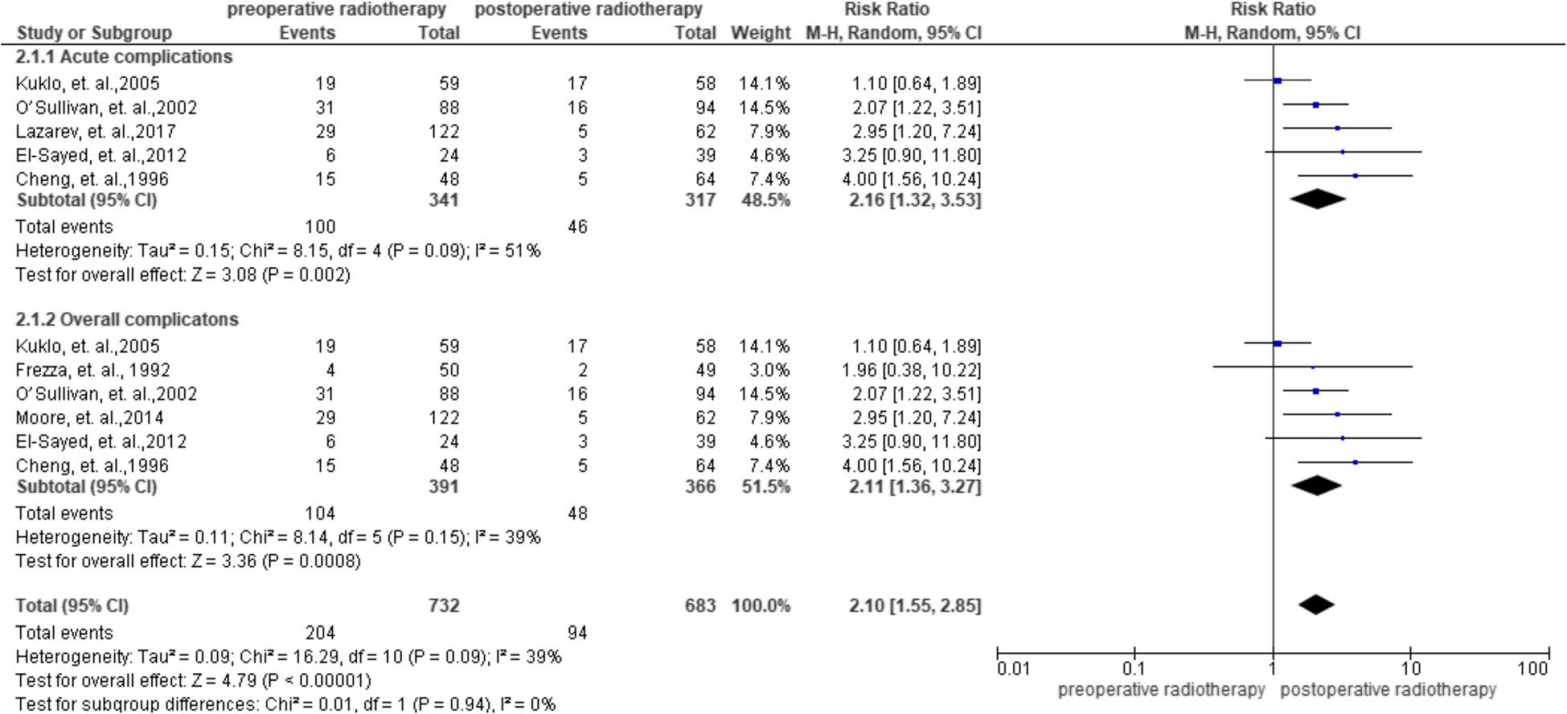
Summarized complications

Of all the 15 studies, 6 studies reported the data on complications and included 391 and 366 patients in the preoperative radiotherapy group and postoperative radiotherapy group, respectively. The results showed a lower heterogeneity across studies with 퐼^2^ = 24%. The RRs in each study ranged from 1.10 to 4.00, and the pooled results revealed that the preoperative radiotherapy group demonstrated the risk for complications with an RR 2.11 (95%CI = 1.36–3.27). Of the six studies that reported data on complications, 5 studies reported acute wound complications and 1 study reported chronic complications. To assess acute and chronic complications of the participants, the acute wound complications was pooled with an RR 2.16 (95%CI = 1.32–3.53). Another study that reported chronic complications in the preoperative and postoperative radiotherapy groups were 4/50 and 2/49 with RR of 1.96 (95%CI = 0.38–10.22). More data on complications was presented in Fig. 3.

### Overall survival

**Fig. 4:**
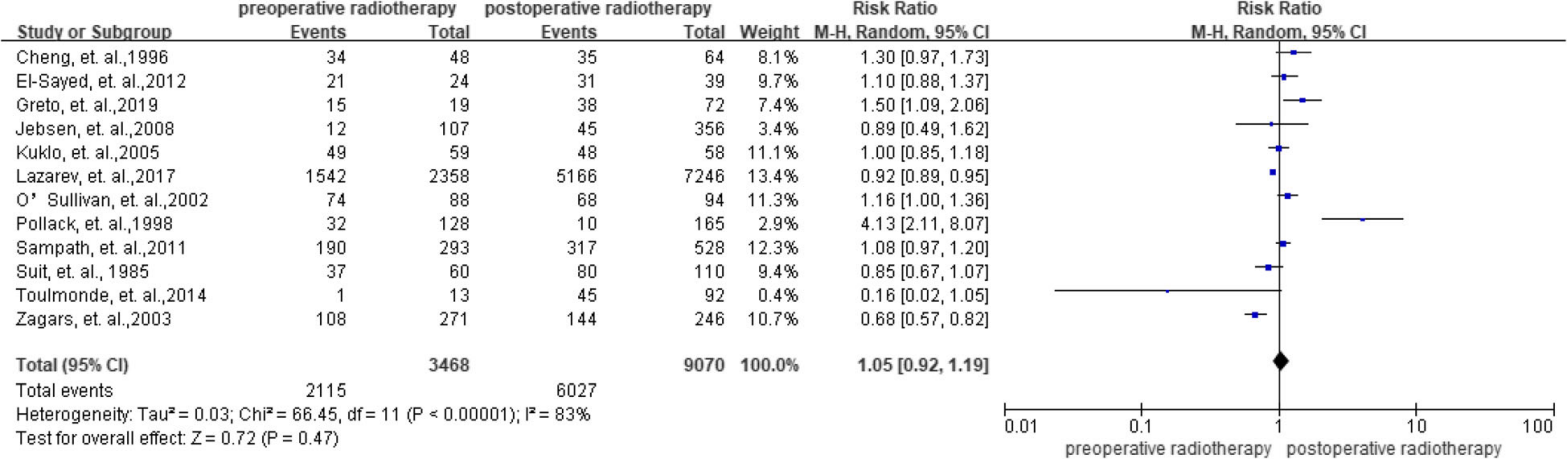
Summarized overall survival

In total, 12 studies investigated the effect of preoperative radiotherapy versus postoperative radiotherapy on overall survival. The meta-analysis across the studies showed no significant benefit with an RR of 1.05 (95%CI = 0.92–1.19) and a higher heterogeneity with an 퐼^2^ = 83% (Fig. 4).

To find the sources of heterogeneity, a sensitivity analysis was done by categorizing the studies into two groups according to the NOS. When the studies with lower quality (i.e., NOS < 7) were removed, the summarized RRs was 0.94 (95% CI = 0.86–1.04) with a significantly decreased heterogeneity of *I*^2^ = 35%. More data was presented in **Additional** Fig. 1.

### Distant metastasis

**Fig. 5:**
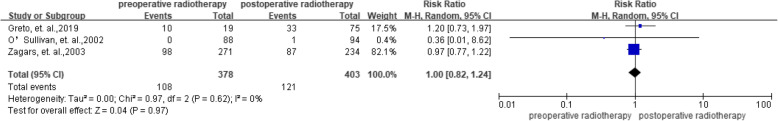
Summarized distant metastasis

As shown in **Figs.****5**, 3 studies included the data on the effect of preoperative radiotherapy versus postoperative radiotherapy on distant metastasis. The pooled results across the studies showed no heterogeneity and no significant benefit with an RR of 1.00 (95%CI = 0.82–1.24).

### Tumor locations

**Additional** Fig. 2**, Additional** Fig. 3**,** and **Additional** Fig. 4 presented stratification analysis on local recurrence, complications, and overall survival of preoperative radiotherapy versus postoperative radiotherapy, respectively. For tumors located at extremities, trunk, head/neck, postoperative radiotherapy demonstrated benefits for local recurrence and overall survival with RR equal to 0.84 (95%CI = 0.62–1.14) and 1.06 (95%CI = 0.92–1.23), respectively. Similarly, for all other locations, the RR for complications was 2.01 (95%CI = 1.22–3.30).

### Publication Bias

No potential publication bias among the included trials was observed according to Begg rank correlation analysis and Egger weighted regression analysis (each *P* value of the analysis was more than 0.05). The detailed potential publication bias for each analysis was presented in **Additional Table****3**.

## Discussion

The current meta-analysis addresses the oncologic outcomes of pre- versus postoperative radiation in patients with resectable soft tissue sarcomas, and 15 studies with 12,813 patients were included for analysis. Most of the included studies had an acceptable quality. Ten studies reported the data on local recurrence and indicated no significance between postoperative radiotherapy and postoperative radiotherapy groups. No difference was observed in the overall survival and distant metastasis between the two groups. According to the pooled result, preoperative radiotherapy group was associated with significant risk for complications.

In most of the recently published meta-analyses on localized resectable soft-tissue sarcomas, 5 eligible studies were included and observed no significant differences in the local recurrence between the two groups with random-effects method [33]. Our study results supported these findings, while another meta-analysis finished in 2018 [10] summarized different results. As the included studies were published in various years and the participants were included with a huge gap, there might be non-uniform standards of disease surveillance among the participating institutions. Also, almost none of the studies matched the participants in the two groups by stage or physical status of the participants. Therefore, selection of patients for preoperative radiotherapy might have been biased towards those with better health. This could be one of the reasons as to why no significant differences were observed. Institutional preference might have included the routine use of preoperative radiotherapy over postoperative radiotherapy, which we were unable to account for. High volume of sarcoma centers might have been preferred for preoperative radiotherapy, and this might bias the results in favor of this group. The most recent data on this topic also supported the point that most of the appreciable increase in preoperative radiation therapy utilization took place in academic facilities [14]. The treatment preference might also bias the results.

In our study, preoperative radiation was associated with higher risk of wound complications than postoperative treatment, and the result was also supported by a recent meta-analysis study [10]. In the current study, six studies reported data on complications and only 5 of these reported acute toxicity. However, in clinical settings, acute toxicity, including surgical wound dehiscence, showed acceptable tolerance and short-term. Therefore, the assessment and comparison of acute and chronic toxicities would be more meaningful in patients. However, due to limited number of studies included, the process cannot be completed in the current study. The assessment and comparison should be highlighted in the future. Local anatomy should be considered when making recommendations on management of an individual patient and a comprehensive suggestion must be considered. When dose and field size issues are considered as the most important criteria, the preoperative approach might be most preferable one. For summarized overall survival and distant metastasis of the two groups, no differences were observed. The results were significantly different from that of the previous meta-analysis study [10]. It was believed that the effect of radiation therapy on overall survival and distant metastasis was decreased in the subtypes that are associated with higher risk of systemic progression (e.g. leiomyosarcoma). However, the conclusion still should be evaluated in a prospective study. Moreover, majority of the participants were treated with postoperative radiotherapy and most of the studies included were retrospective in nature, which might result in selection bias. Fewer participants received combinations of several therapies, such as chemotherapy or locoregional hyperthermia. This is limited by the inclusion of number of the studies and wide range of therapeutic schedules, and so subgroup analysis on specific therapy cannot be performed. Meanwhile, several meaningful subgroup analyses on the outcomes, such as the success of limb salvage process in preoperative and post-operative settings, also could not be completed due to limited number of studies and restricted outcomes. Studies with prospective study design, fraction size, total dose, overall treatment time and/or combination with chemotherapy or targeted agents should be given priority in the future are warranted to increase treatment efficacy and reduce late morbidities [34].

## Conclusion

In conclusion, our meta-analysis provided pooled results that resectable soft tissue sarcoma patients, local recurrence, overall survival, and distant metastasis showed no significant differences between the two therapy methods. However, preoperative radiotherapy group was associated with significant risk for complications when compared with postoperative radiotherapy.

## Supplementary information

**Additional File 1.** Additional Figure 1. Sensitivity analysis on the summarized local recurrence

**Additional File 2.** Additional Figure 2. Summarized local recurrence on extremities, trunk, head/neck sarcoma

**Additional File 3.** Additional Figure 3. Summarized complications on extremities, trunk, head/neck sarcoma

**Additional File 4.** Additional Figure 4. Summarized overall survival on extremities, trunk, head/neck sarcoma

**Additional File 5.** Additional Table 1. Study participants’ characteristics of the included studies

**Additional File 6.** Additional Table 2. Quality assessment of included studies by Newcastle-Ottawa Scale or Jadad scale

**Additional File 7.** Additional Table 3. Publication bias of summarized outcomes

## Data Availability

The datasets used and/or analysed during the current study are available from the corresponding author on reasonable request.

## Abbreviations

RR: Risk ratio
CI: Confidence interval
EBRT: External beam radiation therapy
PRISMA: Preferred Reporting Items for Systematic Reviews and Meta-Analyses
RCTs: Randomized controlled trials
NOS: Newcastle-Ottawa Scale (NOS)
RRs: Risk ratios
CIs: Confidence intervals
