# Supplementary figures and images for: Oncologic outcomes of pre- versus post-operative radiation in Resectable soft tissue sarcoma: a systematic review and meta-analysis
Source: Radiat Oncol. 2020 Jun 23;15:158. doi: 10.1186/s13014-020-01600-9 (PMC7310344; doi:10.1186/s13014-020-01600-9)

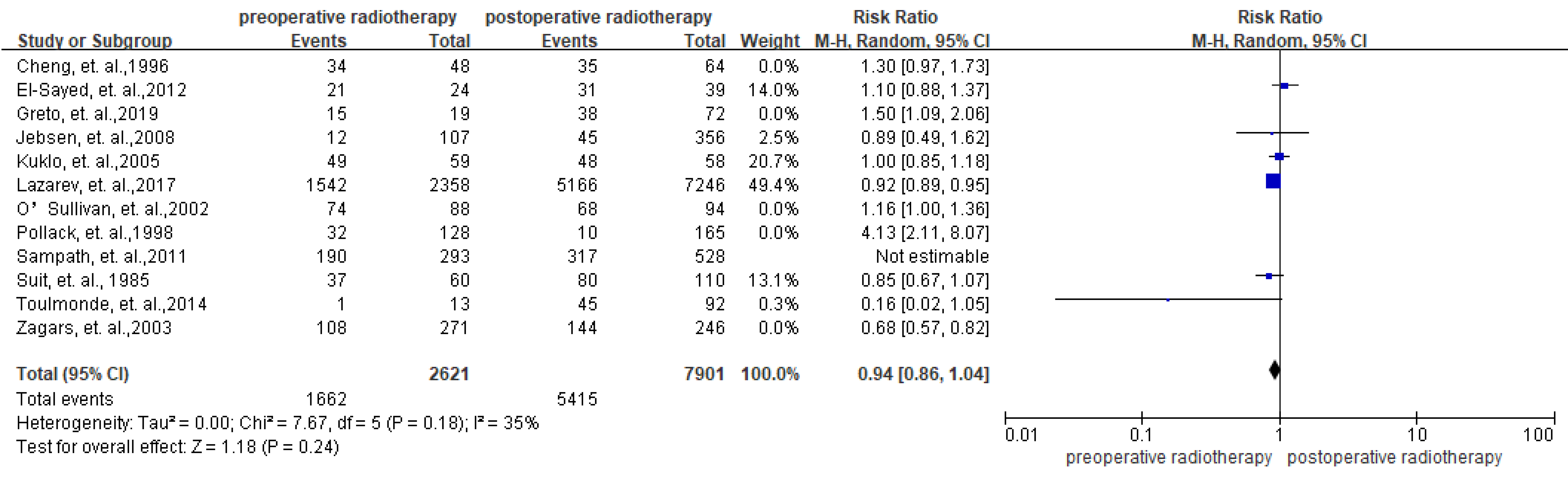

Supplement: Supplementary file 1 — Additional File 1. Additional Figure 1. Sensitivity analysis on the summarized local recurrence [file 13014_2020_1600_MOESM1_ESM.tif]

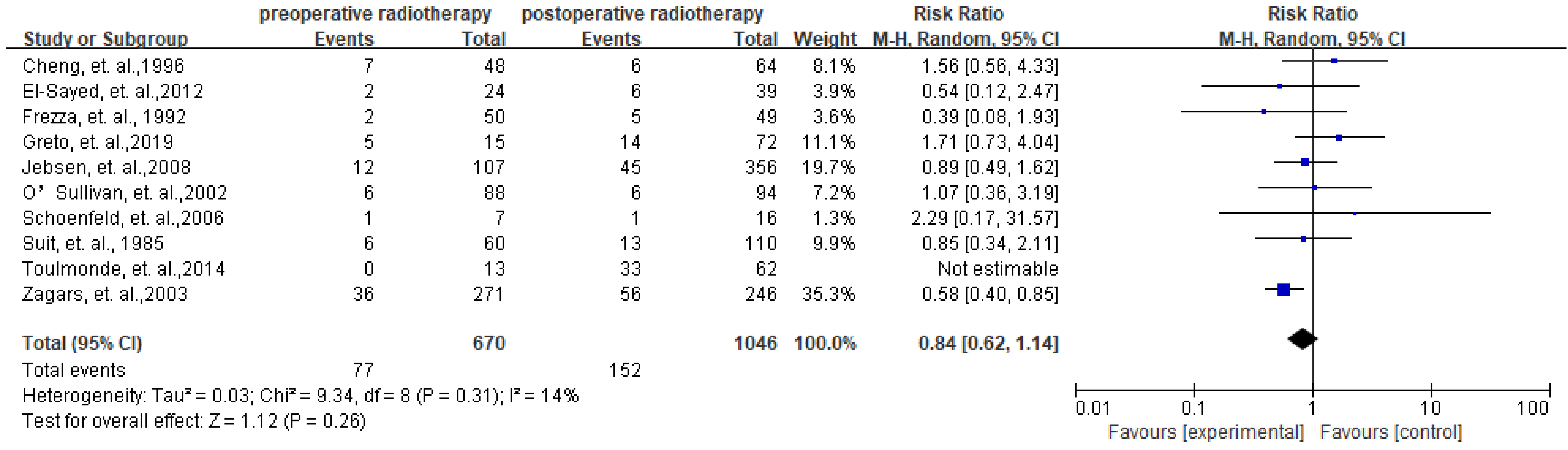

Supplement: Supplementary file 2 — Additional File 2. Additional Figure 2. Summarized local recurrence on extremities, trunk, head/neck sarcoma [file 13014_2020_1600_MOESM2_ESM.tif]

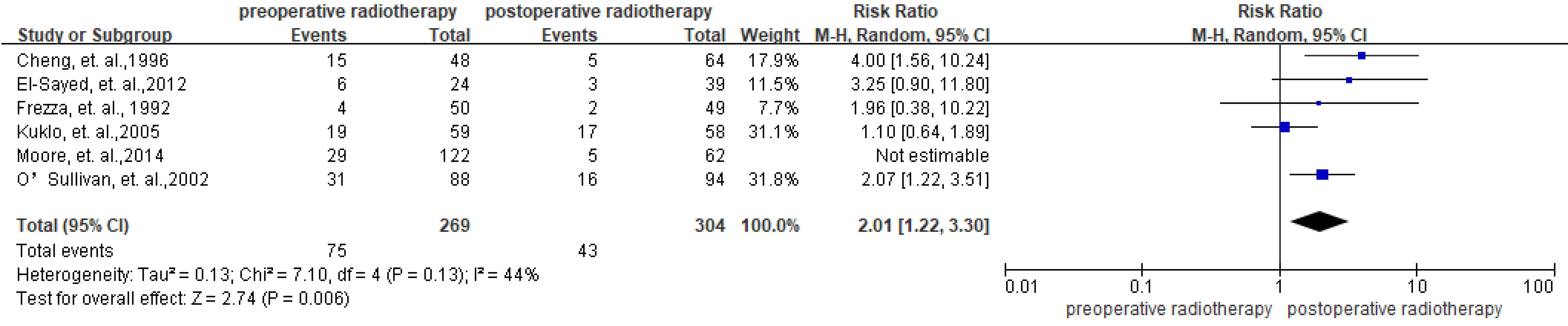

Supplement: Supplementary file 3 — Additional File 3. Additional Figure 3. Summarized complications on extremities, trunk, head/neck sarcoma [file 13014_2020_1600_MOESM3_ESM.tif]

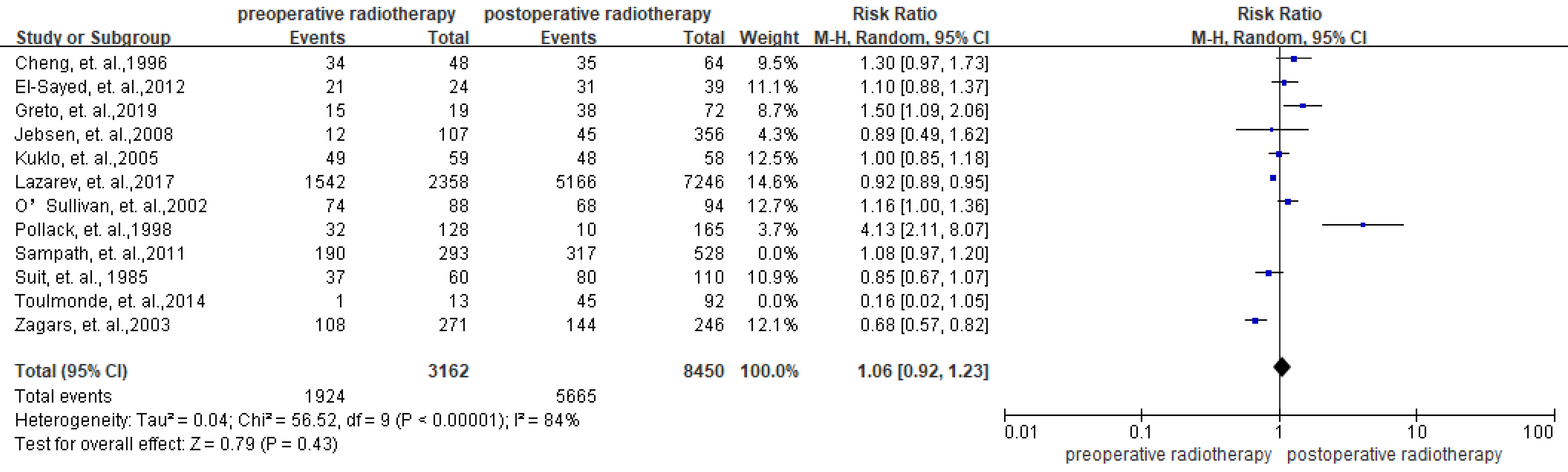

Supplement: Supplementary file 4 — Additional File 4. Additional Figure 4. Summarized overall survival on extremities, trunk, head/neck sarcoma [file 13014_2020_1600_MOESM4_ESM.tif]
