# Supplementary material for: Oncologic outcomes of pre- versus post-operative radiation in Resectable soft tissue sarcoma: a systematic review and meta-analysis
Source: Radiat Oncol. 2020 Jun 23;15:158. doi: 10.1186/s13014-020-01600-9 (PMC7310344; doi:10.1186/s13014-020-01600-9)
Supplement: Supplementary file 5 — Additional File 5. Additional Table 1. Study participants’ characteristics of the included studies [file 13014_2020_1600_MOESM5_ESM.docx]

**Additional Table 1. Study participants’ characteristics of the included studies**

| **Study included** | **Country** | **Study design** | **Study period** | **Gender (% of males)** | **Age (years), means (range)** | **Radiotherapy type** |
| --- | --- | --- | --- | --- | --- | --- |
| Frezza, et. al., 1982[[22](#_ENREF_22)] | United States | Retrospective | 1975-1981 | 76.74 | 47.00 (13.00-80.00) | Preoperative or postoperative external beam radiotherapy |
| Suit, et. al., 1985[[23](#_ENREF_23)] | United States | Retrospective | 1971-1982 | 54.50 | <60: 114.00 (67.00) ^a^  ≥60: 56.00 (33.00) ^a^ | Preoperative, intraoperative or postoperative external beam radiotherapy |
| Cheng, et. al.,1996[[24](#_ENREF_24)] | United States | Retrospective | 1979-1993 | 58.96 | 47.00 (18.00-88.00) | Preoperative or postoperative external beam radiotherapy |
| Pollack, et. al.,1998[[25](#_ENREF_25)] | United States | Retrospective | 1956-1992 | 45.92 | 48.60 (6.00-88.00) | Preoperative or postoperative external beam radiotherapy |
| O’Sullivan, et. al.,2002[[11](#_ENREF_11)] | Canada | RCT | 1994-1997 | 55.00  51.00 | <50: 74.00 (41.00) ^a^  50-70: 71.00(39.00) ^a^  ≥70: 37.00 (20.00) ^a^ | Preoperative or postoperative external beam radiotherapy |
| Zagars, et. al.,2003[[12](#_ENREF_12)] | United States | Retrospective | 1960-1999 | 43.32 | 49.00 (2.00-85.00) | Preoperative, intraoperative or postoperative external beam radiotherapy |
| Kuklo, et. al.,2005[[13](#_ENREF_13)] | United States | Retrospective | 1994-2005 | 45.54 | 38.00 (4.00-72.00) | Preoperative or postoperative external beam radiotherapy |
| Schoenfeld, et. al.,2006[[26](#_ENREF_26)] | United States | Retrospective | 1977-2003 | 43.48 | 64.00 (NA) | Preoperative or postoperative external beam radiotherapy |
| Jebsen, et. al.,2008[[27](#_ENREF_27)] | Norway | Retrospective | 1986-2005 | 51.00 | 60.00 (16.00-95.00) | Preoperative or postoperative external beam radiotherapy |
| Sampath, et. al.,2011[[28](#_ENREF_28)] | Canada | Retrospective | 1984-2005 | 54.00 | 62.00 (18.00-97.00) | Preoperative or postoperative external beam radiotherapy |
| El-Sayed, et. al.,2012[[29](#_ENREF_29)] | Egypt | Prospective | 2006-2012 | 65.10 | 40.00 (19.00-69.00) | Preoperative or postoperative external beam radiotherapy |
| Moore, et. al.,2014[[30](#_ENREF_30)] | Canada | Retrospective | 2000-2011 | 55.86 | 55.00 (9.00–87.00) | Preoperative or postoperative external beam radiotherapy |
| Toulmonde, et. al.,2014[[31](#_ENREF_31)] | France | Retrospective | 1988-2008 | 47.00 | 57.00 (18.00–89.00) | Preoperative or postoperative external beam radiotherapy |
| Lazarev, et. al.,2017[[14](#_ENREF_14)] | United States | Retrospective | 2004-2012 | 56.02 | <60: 4537 (47.24) ^b^  ≥60: 5607 (52.76) ^b^ | Preoperative, intraoperative or postoperative external beam radiotherapy |
| Greto, et. al.,2019[[32](#_ENREF_32)] | Italy | Retrospective | 1991-2007 | 55.86 | 73.00 (66.00-93.00) | Preoperative or postoperative external beam radiotherapy |

Abbreviations: RCT, randomized controlled trial; NA, not available.

^a^, means and percentage.

^b^, number and percentage.
