# Supplementary material for: Oncologic outcomes of pre- versus post-operative radiation in Resectable soft tissue sarcoma: a systematic review and meta-analysis
Source: Radiat Oncol. 2020 Jun 23;15:158. doi: 10.1186/s13014-020-01600-9 (PMC7310344; doi:10.1186/s13014-020-01600-9)
Supplement: Supplementary file 6 — Additional File 6. Additional Table 2. Quality assessment of included studies by Newcastle-Ottawa Scale or Jadad scale [file 13014_2020_1600_MOESM6_ESM.docx]

**Additional Table 2. Quality assessment of included studies by Newcastle-Ottawa Scale or Jadad scale**

| **Study included** | **Overall quality score** |
| --- | --- |
| Suit, et. al., 1985[[23](#_ENREF_23)] | 8 |
| Frezza, et. al., 1992[[22](#_ENREF_22)] | 6 |
| Cheng, et. al.,1996[[24](#_ENREF_24)] | 6 |
| Pollack, et. al.,1998[[25](#_ENREF_25)] | 6 |
| O’Sullivan, et. al.,2002[[11](#_ENREF_11)] | 5 ^a^ |
| Zagars, et. al.,2003[[12](#_ENREF_12)] | 6 |
| Kuklo, et. al.,2005[[13](#_ENREF_13)] | 8 |
| Schoenfeld, et. al.,2006[[26](#_ENREF_26)] | 7 |
| Jebsen, et. al.,2008[[27](#_ENREF_27)] | 8 |
| Sampath, et. al.,2011[[28](#_ENREF_28)] | 7 |
| El-Sayed, et. al.,2012[[29](#_ENREF_29)] | 8 |
| Moore, et. al.,2014[[30](#_ENREF_30)] | 7 |
| Toulmonde, et. al.,2014[[31](#_ENREF_31)] | 8 |
| Lazarev, et. al.,2017[[14](#_ENREF_14)] | 7 |
| Greto, et. al.,2019[[32](#_ENREF_32)] | 6 |

^a^, the Jadad scale and the overall score ranges from 0–5.
