# Supplementary material for: Oncologic outcomes of pre- versus post-operative radiation in Resectable soft tissue sarcoma: a systematic review and meta-analysis
Source: Radiat Oncol. 2020 Jun 23;15:158. doi: 10.1186/s13014-020-01600-9 (PMC7310344; doi:10.1186/s13014-020-01600-9)
Supplement: Supplementary file 7 — Additional File 7. Additional Table 3. Publication bias of summarized outcomes [file 13014_2020_1600_MOESM7_ESM.docx]

**Additional Table 3. Publication bias of summarized outcomes**

| **Outcomes** | **Begg (*P* value)** | **Egger (*P* value)** |
| --- | --- | --- |
| Summarized local recurrence | 0.66 | 0.51 |
| Summarized wound complication | 0.47 | 0.31 |
| Summarized overall survival | 0.28 | 0.16 |
| Summarized distant metastasis | 0.74 | 0.64 |
| Summarized localization stratification analysis of local recurrence | 0.40 | 0.31 |
| Summarized localization stratification analysis of wound complication | 0.21 | 0.11 |
| Summarized localization stratification analysis of overall survival | 0.34 | 0.24 |
